# Linking hypothetical knowledge patterns to disease molecular signatures for biomarker discovery in Alzheimer’s disease

**DOI:** 10.1186/s13073-014-0097-z

**Published:** 2014-12-03

**Authors:** Ashutosh Malhotra, Erfan Younesi, Shweta Bagewadi, Martin Hofmann-Apitius

**Affiliations:** Department of Bioinformatics, Fraunhofer Institute for Algorithms and Scientific Computing (SCAI), Schloss Birlinghoven, 53754 Sankt Augustin, Germany; Rheinische Friedrich-Wilhelms-Universität Bonn, Bonn-Aachen International Center for Information Technology, 53113 Bonn, Germany

## Abstract

**Background:**

A number of compelling candidate Alzheimer’s biomarkers remain buried within the literature. Indeed, there should be a systematic effort towards gathering this information through approaches that mine publicly available data and substantiate supporting evidence through disease modeling methods. In the presented work, we demonstrate that an integrative gray zone mining approach can be used as a way to tackle this challenge successfully.

**Methods:**

The methodology presented in this work combines semantic information retrieval and experimental data through context-specific modeling of molecular interactions underlying stages in Alzheimer’s disease (AD). Information about putative, highly speculative AD biomarkers was harvested from the literature using a semantic framework and was put into a functional context through disease- and stage-specific models. Staging models of AD were further validated for their functional relevance and novel biomarker candidates were predicted at the mechanistic level.

**Results:**

Three interaction models were built representing three stages of AD, namely mild, moderate, and severe stages. Integrated analysis of these models using various arrays of evidence gathered from experimental data and published knowledge resources led to identification of four candidate biomarkers in the mild stage. Mode of action of these candidates was further reasoned in the mechanistic context of models by chains of arguments. Accordingly, we propose that some of these ‘emerging’ potential biomarker candidates have a reasonable mechanistic explanation and deserve to be investigated in more detail.

**Conclusions:**

Systematic exploration of derived hypothetical knowledge leads to generation of a coherent overview on emerging knowledge niches. Integrative analysis of this knowledge in the context of disease mechanism is a promising approach towards identification of candidate biomarkers taking into consideration the complex etiology of disease. The added value of this strategy becomes apparent particularly in the area of biomarker discovery for neurodegenerative diseases where predictive biomarkers are desperately needed.

**Electronic supplementary material:**

The online version of this article (doi:10.1186/s13073-014-0097-z) contains supplementary material, which is available to authorized users.

## Background

Alzheimer’s disease (AD) is an irreversible progressive brain disorder, with an unresolved etiology and characterized by the long latency between the initial dysregulation processes and the late appearance of clinical symptoms [1]. Worldwide, intensive research has been dedicated to investigation of early disease events as early diagnosis of AD is expected to make a big difference in treatment. In particular, an exploration of effective biomarker candidates for identifying pre-symptomatic patients or specific patient subgroups enabling early detection of the disease is necessary [2]. There is currently no validated, FDA-approved biomarker for AD [3]. The almost established (but not yet approved) biomarkers - Tau and APP (fragments) - represent the current diagnostic practice of AD [4,5]. Nonetheless, time course data exist about the appearance of these candidate biomarkers in patient cohorts at different stages and in different risk groups [6]. Sporadic AD is likely to be a complex disease influenced by many factors, which results from various alterations in multiple cellular pathways and processes. These mechanisms offer potential for additional therapeutic targets for the development of new disease-modifying strategies [7]. The AD research community is investigating several promising candidates and the results of such investigations are very often being communicated as a series of reasonable speculations or hypotheses appearing in scientific publications. In order to expand the knowledge space around AD biomarkers, we need to specifically address the so-called ‘gray zone’ that is rich in speculation and hints for emerging knowledge and that exists outside of the well-established world of biomarker candidates such as Tau and APP. A systematic, automated approach towards the identification of hypotheses about proteins playing a role in specific stages of AD mentioned in the scientific literature would be highly desirable.

Previously, Greco *et al.* [8] have successfully demonstrated how text mining and *in silico* analysis can help to derive valid biomarker candidates for AD. However, their analysis covers mining various data sources available until 2006. Moreover, Greco *et al.* have initially used a series of axioms which associate disease and brain regions to proteins expressed in AD without distinction between factual (well established) and speculative (emerging) knowledge. Indeed systematic identification and extraction of emerging biomarker information from the recent literature in the context of the disease condition provides an opportunity to focus on potential candidates with high probability of novelty for further investigation.

A dedicated approach towards harvesting this sort of speculations is a promising try to make use of the collective ‘exploratory intelligence’ in the research community. In particular, hypotheses covering the interaction of genes and proteins in a particular stage of AD could serve as potential diagnostic and therapeutic markers. Recently, we have developed a strategy for the detection of speculative patterns in the scientific literature, named HypothesisFinder [9]. HypothesisFinder is an automated approach that allows for systematic ‘harvesting’ of scientific hypotheses from text sources, particularly in the case of complex, idiopathic diseases, like AD, where the etiology of the disease is still unclear. The approach identifies statements in scientific text that introduce working hypotheses in the following form:

‘Oxidative inactivation of ENO1, GLUL and PIN1 may alter cellular processes and lead to the development of AD from Mild cognitive impairment (MCI).’ (PMID: 16466929)

or

(Biomarker specific hypothetical statement)

‘These results suggest that TACE activity and soluble TNF receptors may be potential diagnostic candidate biomarkers in AD and MCI.’ (PMID: 21978728)

A systematic analysis of all speculative statements of this kind should, therefore, allow us to capture the spectrum of hypotheses existing in the literature about the (molecular) etiology of AD. We have applied this systematic approach to discovery of potential biomarker candidates for AD, with the idea in mind that embedding of information about such putative biomarkers into a bigger disease-specific context should provide more details and explanations on potential mechanisms these candidates may be involved in. In this work, we show how information about putative, highly speculative biomarkers can be enriched and put into a functional context through modeling the molecular interactions and other relationships that these biomarkers could potentially play a role in. Using a network biology approach, we aim to provide an example of how biomarker candidates speculated at the scientific literature could be mechanistically linked to the disease progression. A dedicated protocol presented here allows for the prediction of previously unattended disease-related genes involved in the staging of AD probable of being putative biomarkers and good candidates for further experimental investigation.

A fundamental element of this computational strategy is the combination of knowledge-based and data-based approaches for biomarker prediction [10] as each of the candidates speculated to be biomarkers in the literature (knowledge-based evidences) were further supported by gene expression data (omics-data based evidence). We undertook a systematic approach for the retrieval, extraction and aggregation of independent evidence from the literature reporting on a certain protein being a ‘putative biomarker’. In view of the rapid pace of scientific knowledge published on AD biomarkers, our approach proposes an integrative strategy on how putative biomarkers can be identified and how they can be ‘validated’ by integrating them into a functional context (the mechanistic modeling approaches we undertake). This work demonstrates stage-specific AD models supported by speculations about the potential role of genes/proteins as biomarkers can reveal new insights into putative mechanisms that mark the initiation and progression of the disease.

## Methods

### Domain-specific information retrieval and knowledge extraction

**Figure 1:**
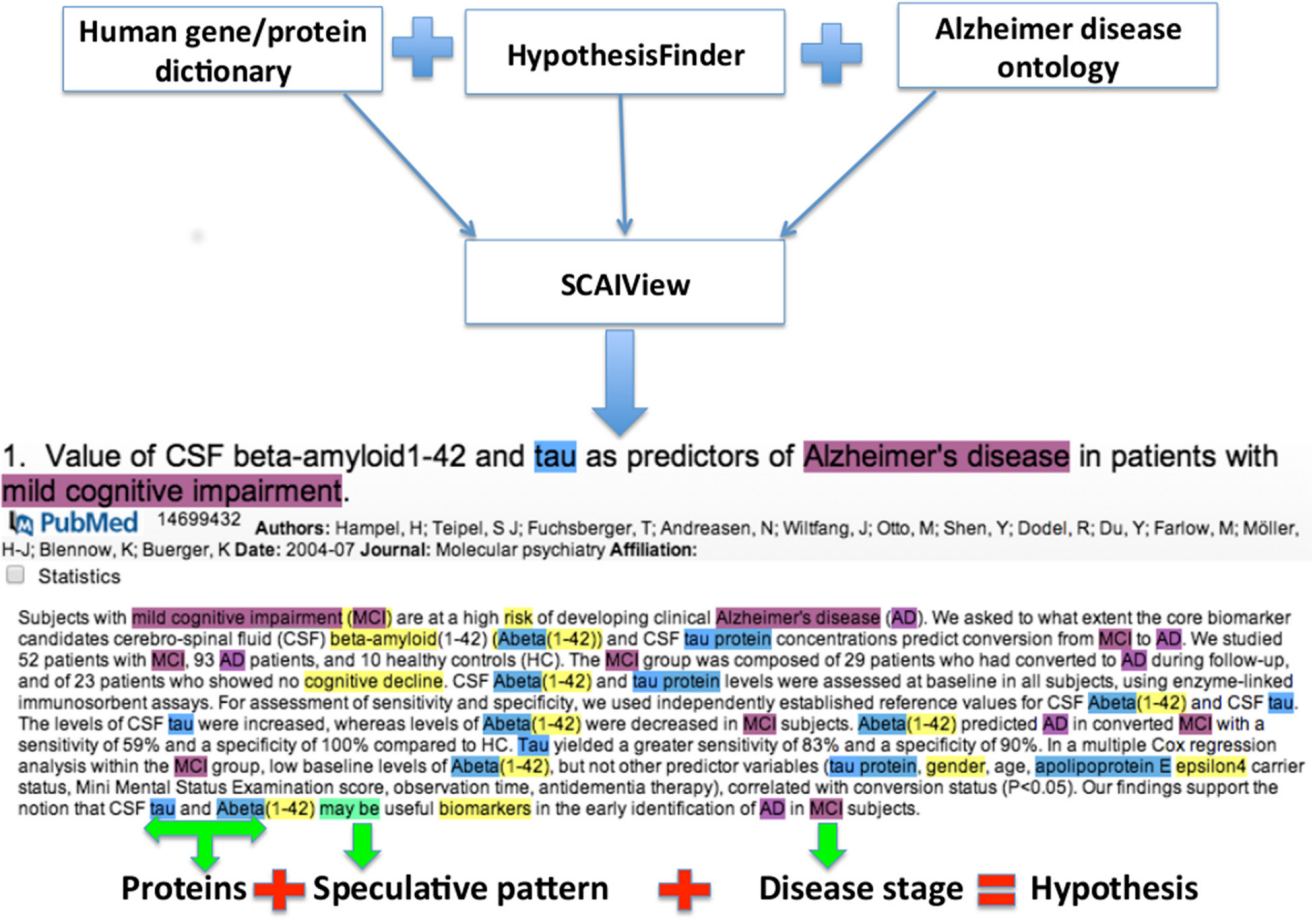
**The document retrieval and information extraction strategy.** Schematic representation demonstrating usage of Human gene/protein dictionary, HypothesisFinder, and ADO integrated in SCAIView for the extraction of hypotheses related to particular stages of Alzheimer's disease from Medline abstracts.

Speculative statements related to human proteins, which define the progression of AD from ‘Mild’ to ‘Severe’ stage, were retrieved from Medline abstracts by using the ‘human gene/protein dictionary’, ‘HypothesisFinder’ [8], and ‘Alzheimer's disease ontology’ (ADO) [11] terminologies within SCAIView [12], a scalable indexing and retrieval platform that has exhibited successful information retrieval scenarios from Medline [13], patents [14], and e-health records [15]. SCAIView supports named entity recognition (NER), information retrieval and information extraction. Querying SCAIView using the intersection of ‘HypothesisFinder’ and ‘human gene/protein dictionary’ makes it possible to capture all speculative sentences related to AD-linked genes/proteins whereas ADO, which contains essential concepts representing stages of AD (Mild, Moderate, Severe) along with all possible synonyms as sub-concepts, allows for stage-specific document retrieval. Using the proposed combinatory approach (Figure 1), we defined a sub-corpus of Medline in SCAIView using the keyword ‘Alzheimer’ and subsequently extracted stage-specific speculative statements from the retrieved documents belonging to the ‘Mild’, ‘Moderate’, and ‘Severe’ stages of AD containing at least one human protein linked to a particular stage of the disease. Extracted hypotheses were manually inspected and checked for their accuracy and relevance to represent one or many processes belonging to a particular AD stage. Statements with no direct link to any stage of AD were discarded. The resulting collection of hypotheses shows a clear relationship to AD stages (‘Mild’, ‘Moderate’, and ‘Severe’) and represents specific events that possibly mark the progression of AD (Additional files 1, 2, and 3).

### Biomarker - specific knowledge extraction

Among the speculative statements retrieved using the above-defined strategy, we were particularly interested in the ones speculating about the possibility that a particular protein (or gene) could be a possible biomarker. The resulting set of speculations pointing at putative biomarkers was manually curated. Additional, evidences for these biomarker candidates were further backed up through dedicated retrieval experiments using the Biomarker terminology [16] within SCAIView. The biomarker terminology helped us to systematically look for evidence (apart from the keyword biomarker) existing in the literature where a particular candidate has been shown to be differentially expressed or have been proven as a genetic marker for AD. This approach - along with manual curation - resulted in a collection of statements describing a role of a particular candidate as a biomarker (Additional file 4) such as:

‘Decreased TrkA gene expression in cholinergic neurons of the striatum and basal forebrain of patients with Alzheimer’s disease.’ (PMID: 9184126)

or

‘Abnormal expression of PPARG protein may correlate with Alzheimer’s disease.’ (PMID: 9920782)

Both speculative and factual statements supporting the candidature of a protein as a potential biomarker were extracted using this approach. Based on the number of evidences (factual + speculative) identified using the described strategy, we were able to distinguish between ‘emerging potential candidates’ whose role in AD is hypothesized till date and ‘known valid potential candidates’, which were also speculated once, but have accumulated substantial evidence gradually with increasing number of evidence appearing in the literature that support the notion that they do in fact have a role in AD progression.

### Construction of stage-specific protein interaction networks

Stage-specific proteins that have been speculated to be involved in the disease staging (as shown in Figure 1) were mapped onto a fully curated Alzheimer-specific protein-protein interaction (PPi) network of 321 nodes and 356 edges (Additional file 5) to generate stage-specific sub-networks. The Alzheimer-specific protein interaction network which serves as a base for the construction of stage specific networks has been derived by mining AD-specific statements from literature (PubMed abstracts + PubMed central documents). Initially a supervised machine-learning approach designed by Bobic *et al.* [17] was used to automatically extract sentences existing in the literature where two proteins have been mentioned to interact with each other. Such evidence were further manually filtered by three annotators following strict annotation guidelines (Additional file 5) looking typically for relationships existing in the literature where two proteins have been shown to interact physically with each other. These annotators individually annotated each and every protein interaction evidence and their annotations were used to calculate the inter-annotator agreement (IAA). The IAA determines the quality and acceptability of the notion among annotators and provides a rationale for measuring the quality of prior developed annotation guidelines. The kappa value between the annotators was calculated as high as 0.81, which indicates an acceptable agreement, given the complicated nature of the annotated evidences.

Interactions (edges) of the derived AD network are directed and annotated with entities like *type_of_experiment_performed* (which can have two values: *InVivo* or *InVitro*) to confirm an interaction. Only the connected components of the sub-networks were taken into consideration for further analysis, as only they provide a chance for an embedding into a functional context and a possible mechanistic insight. The Cytoscape environment was used for visualization, construction, and analysis of the stage-wise sub-networks [18].

### Heterogeneous data integration for biomarker discovery

To identify a set of candidate biomarkers for AD, we combined knowledge-driven and data-driven methods [10]. More specifically, a knowledge-driven approach is based on experts’ opinions and knowledge published in the biomedical literature, while a data-driven approach is solely based on the observational data (in this case, gene expression analysis). To generate supportive evidence for the functional relevance of the predicted candidates, knockout mouse phenotypes of the gene of interest were retrieved from MGI database [19]. A putative biomarker was considered ‘more relevant’ or ‘more promising’ when a knockout mouse phenotype provided supportive evidence that the gene or protein speculated to be a biomarker displayed a phenotype that would be in line with the putative role of that gene or protein in the pathophysiology process modeled.

### Cause-and-effect knowledge models

In an attempt to provide a clue to the potential mechanism underlying the role and functional involvement of a putative biomarker, we tried to embed the biomarker candidate into a model representing causal and correlative relationships. The modeling syntax used for this is OpenBEL, an open access version of the Biological Expression Language (BEL) [20]. Each candidate predicted in the course of this work is described by a BEL model embedding the candidate biomarker in a network of causal and correlative relationships. This embedding in a bigger context provides a means for dedicated approaches aimed at the identification of disease mechanisms. Evidences existing in the literature that lend support to the role of a candidate in disease context were encoded in BEL language v1.0 and used to build the BEL model. Each BEL model was validated for correct syntax and compiled using the OpenBELFramework v2.0. The models were visualized using Cytoscape and queried using the OpenBEL KAM Navigator Cytoscape plug-in [21].

### Microarray data extraction

To support exploratory analysis of the proposed biomarkers, AD-related microarray experiments were manually selected from both GEO and ArrayExpress [22,23]. Three experiments were retrieved (GEO IDs: GSE1297, GSE12685, and GSE28146) when searched using the keywords ‘incipient Alzheimer’, filtered for organism ‘*Homo sapiens*’. Additionally, these experiments contained stage specific brain samples. GSE1297 experiment was performed on hippocampal CA1 tissue obtained from healthy, incipient, moderate, and severe stage AD patients. For similar stage information, GSE28146 experiment was carried out on laser-captured hippocampal CA1 tissue gray matter. On the other hand, GSE1297 experiment contains frontal cortex synaptoneurosome samples from incipient AD patients. Statistical data analysis was performed using ‘R’ [24]. The data were normalized using Robust Microarray Average (RMA) [25]. Differentially expressed genes were identified by unpaired t-test across each stage and control samples using the *limma* package [26]. The genes were ranked based on the *P* value of their differential expression, with a cutoff of 0.05*.*

To summarize, our computationally driven, integrative approach concatenates:

- Information retrieval and information extraction from scientific text;
- Information about disease-associated proteins and their interactions;
- Information about hypothetical biomarker candidates and their potential functional involvement in disease progression;
- Information about candidates and their role as potential biomarker;
- Information about AD-specific, quality-controlled gene expression data.

**Figure 2:**
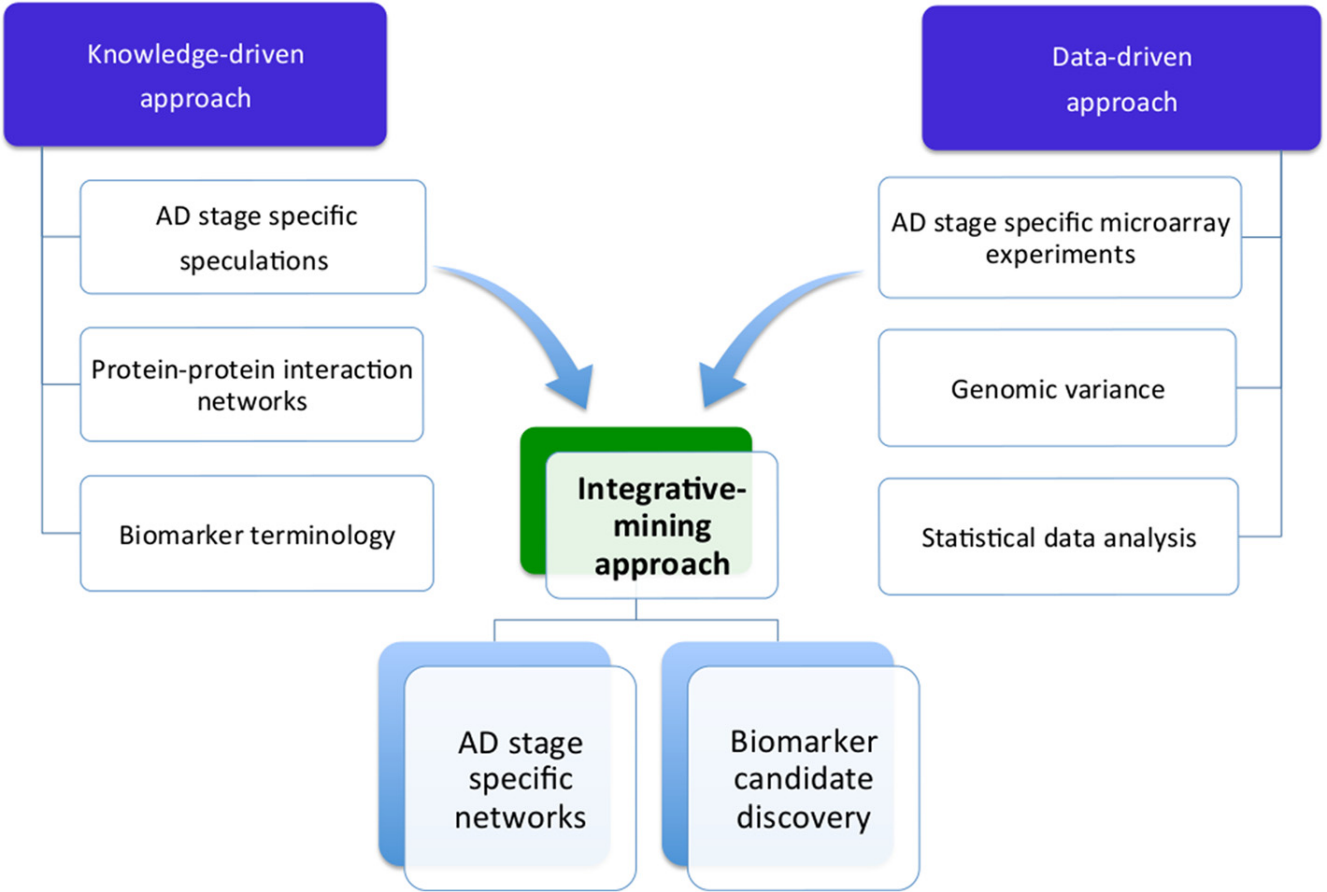
**The integrative-mining strategy.** The approach combines biomedical knowledge and data into a single disease model and makes them jointly amenable for mining and interpretation by experts in the field.

An overview of the methodology described in this section is provided in (Figure 2).

## Results

### Classification of hypothetical statements and their corresponding molecular entities

**Figure 3:**
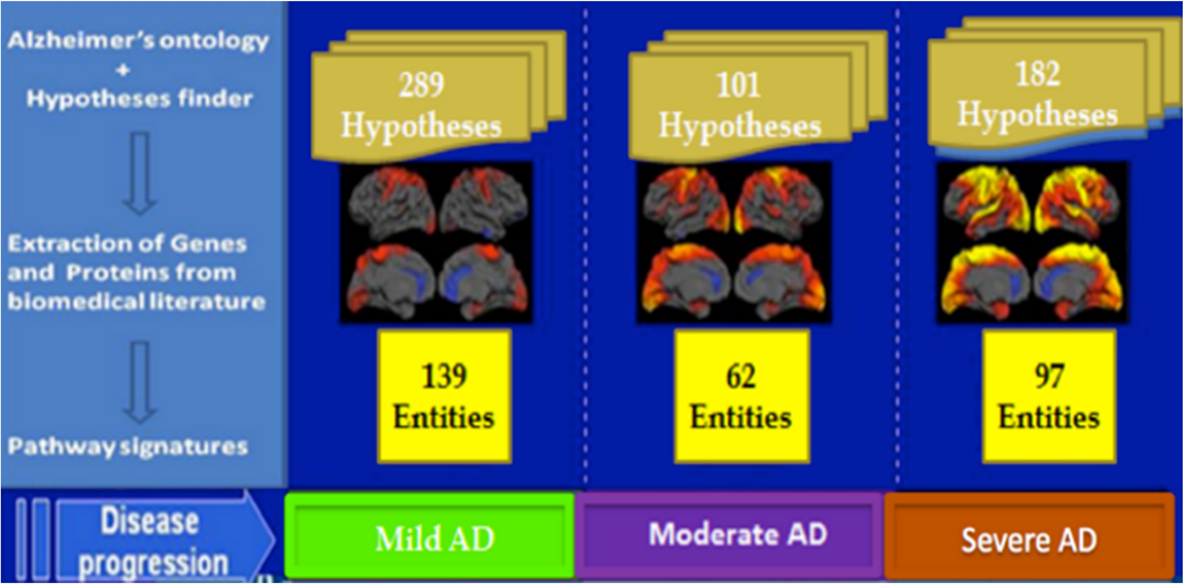
**Correlation of molecular hypotheses to AD staging.** Proteins involved in AD represented as entities related to hypotheses present in literature linked to disease progression.

Using the combination of HypothesisFinder, the ADO, and the Gene/Protein detection system in SCAIView (see the methodology section), we extracted 289 speculative biomarker statements for mild, 101 for moderate, and 182 for severe stages of AD from a filtered corpus of 57,345 AD-related abstracts gathered from Medline [27]. Along with the number of hypotheses, our approach also identified several proteins (Mild: 139, Moderate: 62, Severe: 97) linked to these hypotheses that have been speculated to play a role in a particular stage of AD (Figure 3).

### Analysis of hypothetical sub-networks

**Figure 4:**
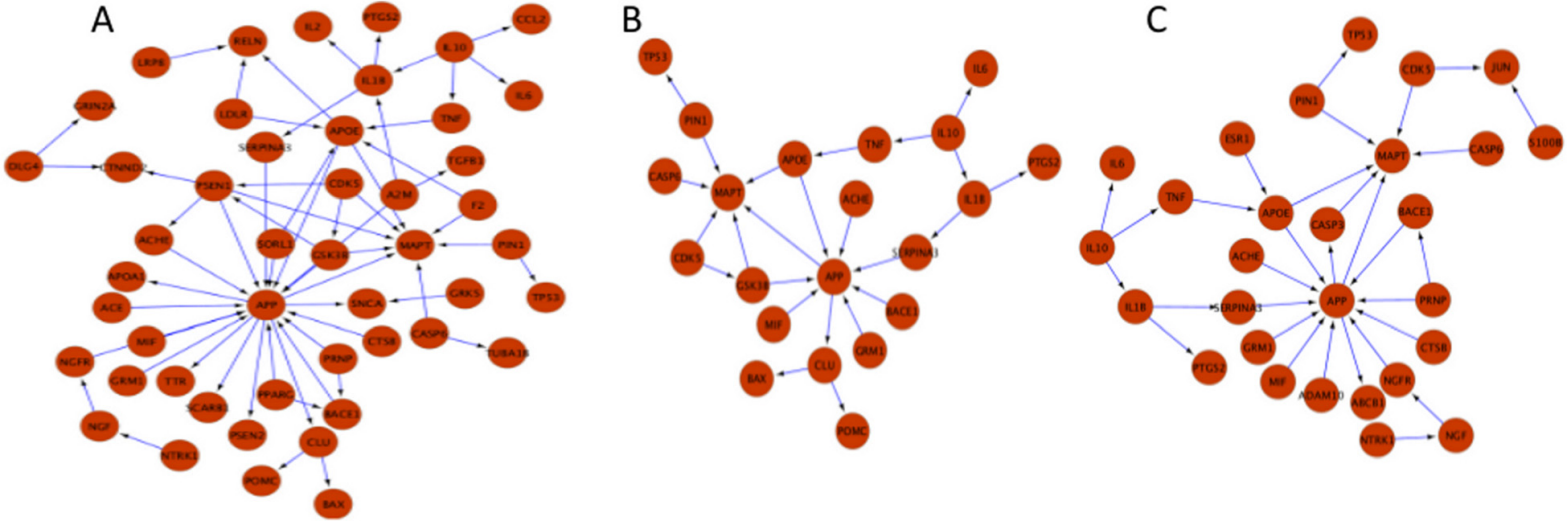
**Stage-specific AD networks.** Protein interaction networks for proteins hypothesized to play a role in Mild **(A)**, Moderate **(B)**, and Severe **(C)** stages of AD.

Mapping of proteins associated with the extracted stage-specific hypotheses onto the AD-specific network (see Methods) resulted in the generation of three stage-specific sub-networks. (Figure 4, Table 1) [28].

**Table 1:** **Statistics of stage specific AD sub-networks**

**Stage**	**Nodes (proteins) (n)**	**Edges (interactions) (n)**
Mild	61	68
Moderate	29	28
Severe	40	37

Here, nodes correspond to proteins whose interaction during AD progression is shown by edges.

As Table 1 indicates, the Mild stage network is the one with a maximum representation of proteins hypothesized to be involved in AD. This is most likely due to the fact that the initial causes for AD are unknown; therefore, researchers are intensively speculating about possible mechanisms that could lead to the defined symptoms. An overlay of 46 literature-derived putative AD biomarkers on the mild stage sub-network resulted in a 100% superimpose showing that this sub-network is highly enriched for the presence of the majority of reportedly altered proteins under AD condition as all of the potential candidates biomarkers mapped onto this network.

### *In silico* discovery of candidate biomarkers

Since AD starts developing 15 to 20 years before signs and symptoms appear [29], an early and efficient diagnosis of the disease is important not only to rule out non-AD pathology but also to identify the best treatment strategies. Hence, we made an attempt to explore novel candidate options particularly in the early stage.

**Figure 5:**
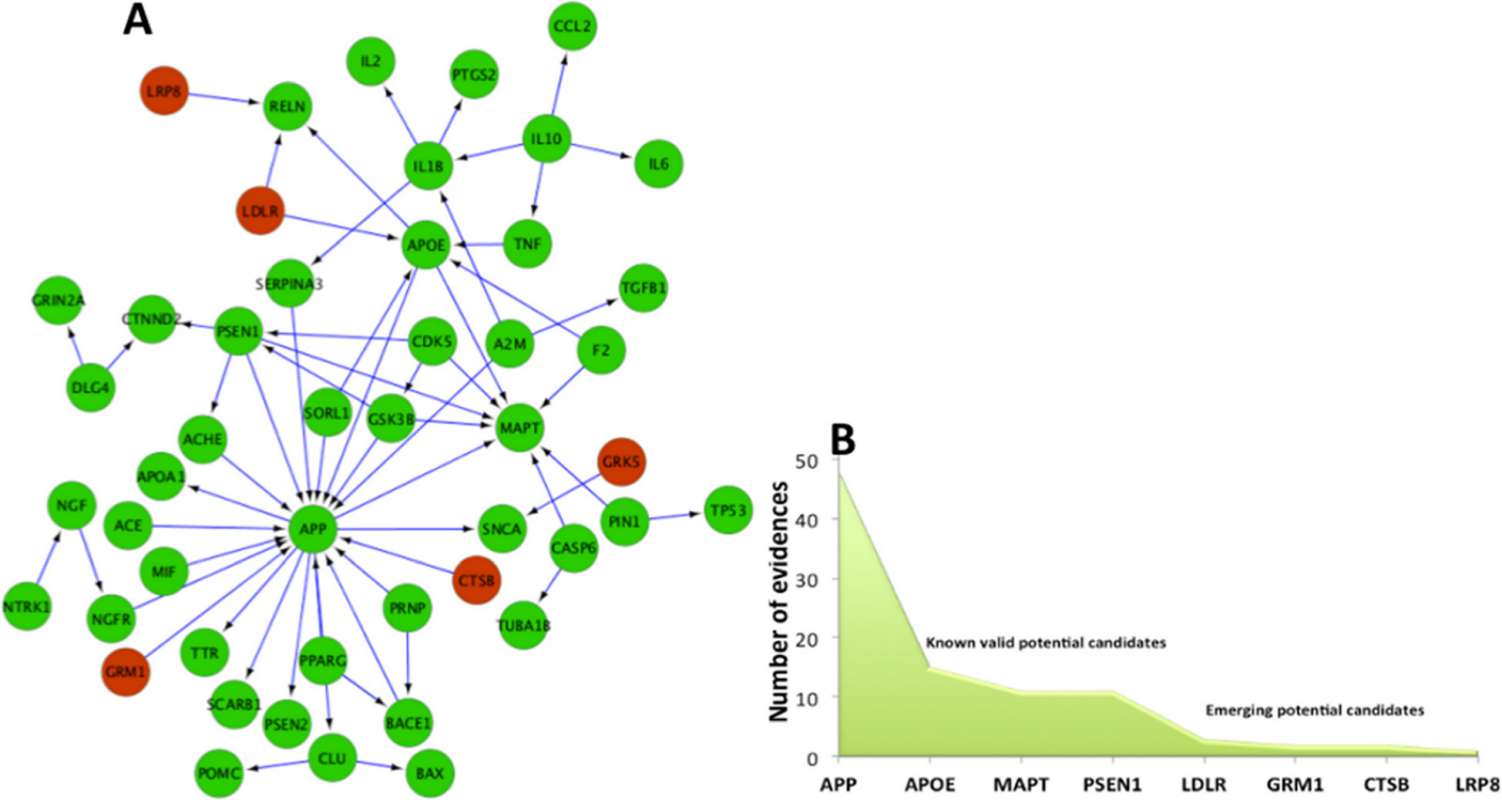
**Network of biomarker candidates presumed to play role in mild AD. (A)** Protein interaction network for Mild AD where green nodes represent ‘known valid potential biomarker’ candidates extracted by literature mining and red nodes serve as new ‘emerging’ candidates. **(B)** Distribution showing the number of evidence extracted from literature, which support the role of a particular candidate as a biomarker for AD.

After mapping the biomarker candidates reported in the literature to be differentially expressed or genetically related to AD (see the section Biomarker-specific knowledge extraction in Methods) on to the mild-stage AD network (Figure 5A) it was found that ‘emerging potential candidates’ whose role in AD is, so far, highly speculative, could be distinguished from ‘known valid potential candidates’, which were also speculated to be potential biomarkers in the past, but have been confirmed to be strongly associated with AD progression according to the literature published in the following years (Figure 5B).

Comprehensive analysis of the early stage network for proteins that are supported by the number of literature-derived evidence revealed that five candidates (LRP8, GRM1, CTSB, GRK5, LDLR) are highly speculated as biomarkers in the literature in terms of their novelty in the context of core AD pathomechanism so that they are altogether supported by 17 speculative statements. In order to further validate this ‘gray zone mining approach’, we checked the expression profile of each candidate under disease condition with publicly available gene expression data. Stringent filtering of relevant expression data sets identified three experiment series relevant to mild stage AD: GSE1297, GSE12685, and GSE28146. Among these, GSE12685 analysis recovered four out of five proposed biomarker genes, LRP8, CTSB, GRM1, and LDLR, to be differentially expressed in frontal cortex synaptoneurosome (Table 2). The former two genes are upregulated and the latter two are downregulated implying that these particular candidates may play a role in early AD.

**Table 2:** **Differential expression values of the speculated potential biomarker candidates in GSE12685**

**Speculated potential biomarker candidates**	**logFC**	**Regulation type**
LRP8	0.199	Upregulated
GRM1	0.235	Downregulated
CTSB	0.367	Upregulated
LDLR	0.446	Downregulated

To show the relevance of these gene expression regulation events to the mechanistic mode of action in our models, we modeled the functional context around these biomarker candidates using the Biological Expression Language (BEL), which integrates literature-derived ‘cause and effect’ relationships into network models. We created individual models specific to each putative biomarker candidate; these biomarker candidate models can also be integrated with other BEL-based models and the functional context, in which the biomarker candidates are being represented and can be extended through this integration. The models provide chains of argumentation for the causal effects and cover a broad spectrum of events that lead to clinical readouts often seen in AD patients. For the sake of better understandability of the context and visualization, the BEL based biomarker candidate models [28] were converted into cartoon-like representations to facilitate interpretation by biologists.

**Figure 6:**
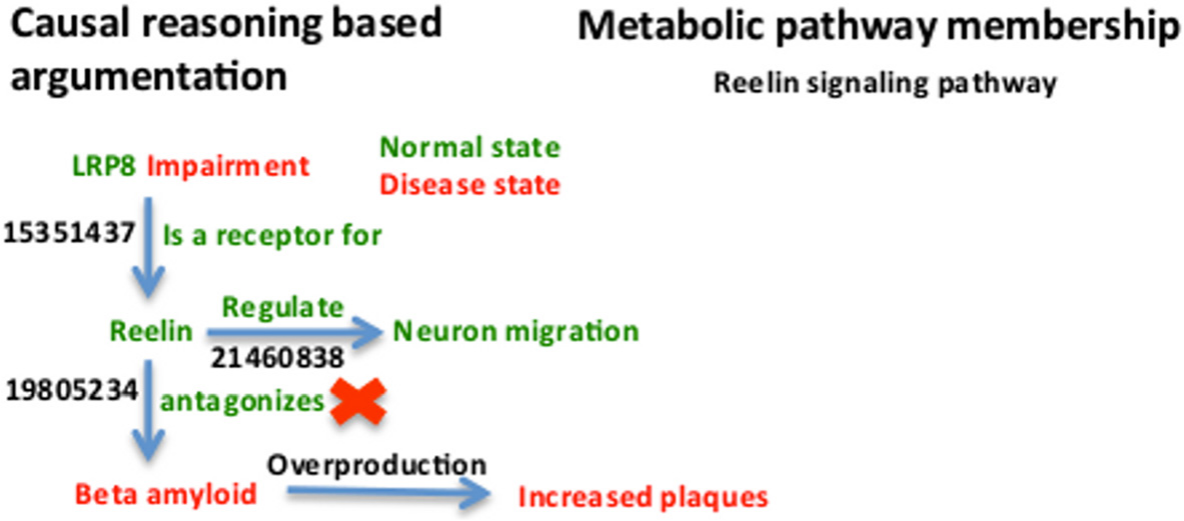
**Cartoon-like representations of putative biomarker: Low-density lipoprotein receptor-related protein 8 (LRP8).** The model illustrates the functional role of the proposed biomarker candidate at a mechanistic level in mild stage of AD supported by PubMed identifiers at edges.

Modeling the ‘chains of causal relationships’ around the putative biomarker candidates provided new insights into their potential mechanistic involvement. One of the advantages of this approach is that the biological mechanism is supported by additional context around an initial hypothesis. For instance, Sagare *et al.* have proposed LRP8 as a biomarker in 2010 [30] based on the finding that in AD, there is increased expression on LRP8. Authors of this study also proclaimed that, in spite of increased level of Lipoprotein receptor protein, it does not bind to amyloid beta (contrary to normal condition), thereby leading to increased concentrations of free amyloid beta and contributing to the disease development. However, our mechanistic model extends beyond this explanation and shed light on the role of ‘Reelin’ - an intermediate molecule linking LRP8 to amyloid beta (Figure 6).

LRP8 is a receptor of Reelin and, in its normal state, Reelin antagonizes amyloid beta production [31]. Meanwhile, in AD there is increased expression of LRP8, which as per our model alters the antagonizing function of Reelin possibly resulting in excess release of amyloid which may aggregate as plaques.

**Figure 7:**
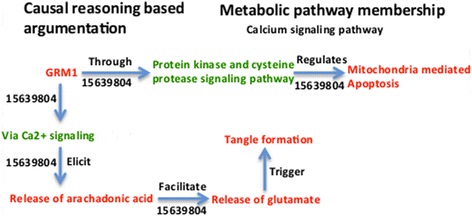
**Cartoon-like representations of putative biomarker: Glutamate receptor, metabotropic 1 (GRM1).** The model illustrates the functional role of the proposed biomarker candidate at a mechanistic level in mild stage of AD supported by PubMed identifiers at edges.

Similarly, we reasoned over interlinked molecules and processes around GRM1 by combining evidence scattered in the literature (Figure 7). We argue that decreased expression of GRM1 elicits release of arachidonic acid via intracellular Ca2+ mobilization, further facilitating glutamate-dependent tangle formation [32]. Also, evidence suggests that GRM1 plays a role in specific signaling pathways thereby regulating ‘mitochondria mediated apoptosis’, a mechanism associated with early AD [33].

**Figure 8:**
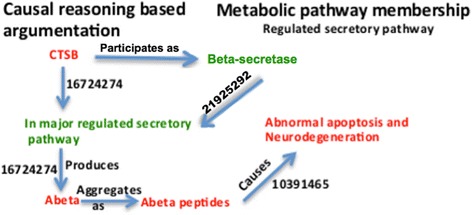
**Cartoon-like representations of putative biomarker: Cathepsin B (CTSB).** The model illustrates the functional role of the proposed biomarker candidate at a mechanistic level in mild stage of AD supported by PubMed identifiers at edges.

The regulated secretory pathway of neurons is the major source of toxic beta-amyloid peptides that accumulates in AD [33]. Extracellular beta-amyloid peptides secreted from this pathway are generated by beta-secretase processing of amyloid precursor protein [34]. Higher CTSB level has been reported in AD patients [35] and it participates as beta-secretase in secretory vesicles of neuronal cells [35] (Figure 8). Cathepsin B stimulates beta-secretase cleavage of APP [36], resulting in higher levels of Abeta peptides which aggregates as plaques. These literature-based arrays of evidence supporting role of CTSB in early AD indicate the mechanisms forming the core of the hypothesis in which CTSB has been speculated as a biomarker for AD.

**Figure 9:**
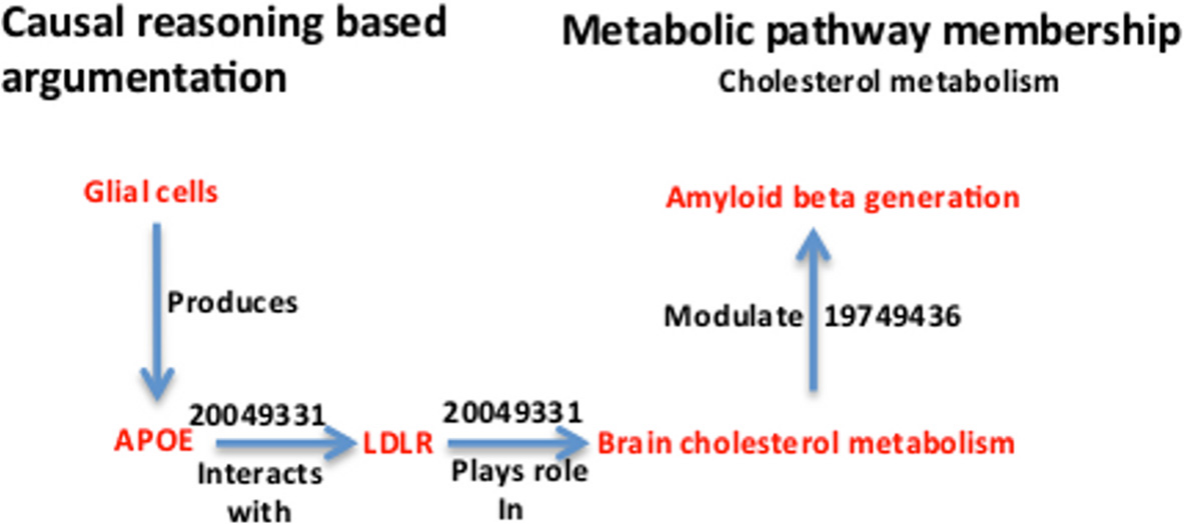
**Cartoon-like representations of putative biomarker: Low density lipoprotein receptor (LDLR).** The model illustrates the functional role of the proposed biomarker candidate at a mechanistic level in mild stage of AD supported by PubMed identifiers at edges.

As yet another speculative context that bears the potential for a new candidate biomarker (Figure 9), we present the involvement of LDLR in brain cholesterol metabolism serving as a putative upstream controller with downstream effects on amyloid beta peptide generation. Low-density lipoprotein receptor (LDLR) binds to ApoE protein and could dramatically affect the development of AD. LDLR plays a key role in cholesterol metabolism in the periphery by facilitating the removal of cholesterol-containing lipoprotein particles from the circulation [37]. The uptake of lipoprotein particles occurs through the binding of ApoE to LDLR [37]. Intracellular cholesterol metabolism was reported to modulate amyloid-beta (Abeta) generation in AD [38]. An increased level of LDLR protein has been shown to dramatically decrease toxic amyloid deposition in the brain [39] and vice versa experiments with cell cultures models of AD in both human and mouse show that increased expression of APP and Abeta downregulate LDLR expression [40]. Also, based on ADNI data it has been shown that a polymorphism (rs5930) in LDLR locus is highly associated with AD in various population cohorts [41].

Further, supportive evidence confirming the role of these candidates in the disease development came from the systematic analysis of complementary data form functional effect of knockouts (Table 3). Based on computational BEL models generated during the course of this work, the mode of action of putative biomarkers has been mechanistically linked to the pathology of AD using literature-derived evidence. However, to additionally support the functional context of these candidates in relation to the nervous system *invivo*, knock out phenotypes were used as supportive evidence. By this means, we check for the plausibility whether experimentally derived explanations by BEL models can be ‘validated’ (enriched with supportive evidence) by animal models.

**Table 3:** **Knockout mouse phenotypes observed for the predicted, speculative biomarker candidates**

**S No,**	**Name of the candidate**	**Mutation category**	**Observed effect on the nervous system**
1	LRP8	Targeted (knockouts)	Abnormal neuronal migration, abnormal brain development abnormal rostral migratory stream morphology, abnormal hippocampus development
2	CTSB	Targeted (knockouts)	Abnormal neuron apoptosis, abnormal hippocampus pyramidal cell layer gliosis neuron degeneration, Purkinje cell degeneration
3	GRM1	Targeted (knockouts)	Reduced long-term potentiation, reduced long-term depression
4	LDLR	Targeted (knockouts)	Abnormal CNS glial cell morphology, abnormal microglial cell morphology, abnormal astrocyte morphology, amyloid beta deposits, abnormal synaptic bouton

## Discussion

There is an unmet need for strategies to model and find potential biomarkers for the identification of early disease-initiating events/mechanisms. In particular, in complex diseases like AD, rather than sticking to the ‘well-known’, we should aim at integrating both established and emerging knowledge in integrative models. Linking hypotheses to the established knowledge or background theory can strengthen the ability of hypothesis-driven predictions. Encoding relevant knowledge into causal relationship models confers enhanced interpretation power that is well suited to back up existing hypotheses. In this work, we demonstrate a novel approach to systematically identify biomarker candidates, which are emerging in the literature. We show, how some of these candidates can be embedded into a functional context by mapping them into a network model representing causal and correlative relationships.

We are aware that we ‘increase the level of speculation’ when we generate these ‘speculative functional context models’, but at the same time, we generate testable hypotheses through this modeling and mining approach. We believe that the mining and modeling of putative biomarker candidates contribute to the rationalization of biomarker discovery projects in AD and in particular helps to come up with hypotheses on ‘early’ putative disease mechanisms.

We make use of linguistic and textual analysis patterns to comprehensively analyze the biomedical literature. Based on the extracted evidences we separated ‘known valid potential candidates’ who have been extensively studied and now have been confirmed to play a role in AD like APP, APOE, MAPT, and so on from ‘emerging potential candidates’ whose role in AD is hypothesized until today. After profound analysis of evidences gathered from various data resources, some of the ‘emerging’ potential candidates, in particular four proteins (LRP8, CTSB, GRM1, LDLR) deserve to be investigated in more detail for their potential to serve as probable biomarkers for early detection of AD. We do acknowledge that the computational modeling results presented here are precursors to clinical and laboratory research findings. However, the predicted, putative biomarker candidates enriched with supporting evidences may guide future validation efforts in experimental clinical research and molecular biology laboratories.

The advantage of following such *in silico* approaches is that they maximize ‘recall’ (the completeness of relevant information) and thus reduce the chance of missing out important information, which may not be easy to assemble following traditional, empirical procedures. Systematic collection of hypotheses allows for rationalization of discussions about possible interpretation of data. Exploration of hypotheses provides an overview on emerging knowledge niches, which have the potential to add value to ongoing research activities. Since speculations represent the gray zone of scientific knowledge, they can provide incremental support to the main hypothesis underlying the research. Conversely, if the speculations are contradictory, then they could shift the direction of the research towards new and rewarding avenues.

The proposed approach for emerging biomarker prediction supports the notion that integration of experiment- and literature-driven information into a single model can be instrumental for prediction, analysis, and interpretation of possible biological mechanisms underlying a disease process. Using this approach, we could demonstrate that emerging potential biomarker candidates (LRP8, GRM1, CTSB, LDLR) can be identified and put into a meaningful functional context that certainly merits further investigation.

## Conclusion

Published speculations identifying potential biomarkers when backed up by molecular mechanisms underlying normal and diseased biological processes, provide valuable input for the generation of new research ideas. In the future, we intend to build highly curated disease models enhanced with the so-called ‘gray zone information’. In particular, in complex diseases like AD, rather than sticking to the ‘well-known’, we should aim at integrating both established and emerging knowledge in integrative models. Encoding relevant knowledge (emerging + established) into causal relationship models confers enhanced interpretation power that is well suited for screening of the most suitable candidates to further advance biomarker discovery and development efforts.

## Additional files

Additional file 1:
**Hypothesis related to Mild stage of AD extracted by using HypothesisFinder in combination with Alzheimer’s disease ontology (ADO) and Human gene and protein dictionary within SCAIView.**
Additional file 2:
**Hypothesis related to Moderate stage of AD extracted by using HypothesisFinder in combination with Alzheimer’s disease ontology (ADO) and Human gene and protein dictionary within SCAIView.**
Additional file 3:
**Hypothesis related to Severe stage of AD extracted by using HypothesisFinder in combination with Alzheimer’s disease ontology (ADO) and Human gene and protein dictionary within SCAIView.**
Additional file 4:
**Evidences extracted from literature using biomarker terminology where particular candidates have been mentioned as Biomarkers for AD.**
Additional file 5:
**Description of annotation guidelines followed to construct a PPi network of AD by gathering evidences existing in literature.**
