# Supplementary material for: Linking hypothetical knowledge patterns to disease molecular signatures for biomarker discovery in Alzheimer’s disease
Source: Genome Med. 2014 Dec 3;6(11):97. doi: 10.1186/s13073-014-0097-z (PMC4256903; doi:10.1186/s13073-014-0097-z)
Supplement: Additional file 4: — Evidences extracted from literature using biomarker terminology where particular candidates have been mentioned as Biomarkers for AD. [file 13073_2014_97_MOESM4_ESM.docx]

CASP6

- increased expression of CASP6 in frontal cortex correlates with increased apoptosis associated with Alzheimer disease [PMID: 15277226 ]
- increased expression of CASP6 in frontal cortex correlates with nerve degeneration associated with Alzheimer disease [PMID: 15277226 ]
- increased expression of CASP6 in temporal cortex correlates with increased apoptosis associated with Alzheimer disease [PMID: 15277226 ]
- increased expression of CASP6 in temporal cortex correlates with nerve degeneration associated with Alzheimer disease [PMID: 15277226 ]
- mislocalization of CASP6 protein correlates with nerve degeneration associated with Alzheimer disease [PMID: 15277226 ]

TNF

- increased expression of TNF in blood correlates with Alzheimer disease [PMID: 14975597 ]
- increased expression of TNF in cerebrospinal fluid correlates with Alzheimer disease [PMID: 15936505 ]
- increased expression of TNF in cerebrospinal fluid correlates with early stage or low grade form of Alzheimer disease [PMID: 11164279 ]
- increased expression of TNF in microglia may correlate with inflammation associated with Alzheimer disease [PMID: 11424194  , 12404514 ]
- increased expression of TNF in serum correlates with increased T cell activation associated with Alzheimer disease [PMID: 12493553 ]
- polymorphism in the TNF gene correlates with increased occurrence of disease susceptibility associated with Alzheimer disease [PMID: 11121190 ]
- polymorphism in the TNF gene correlates with late onset form of Alzheimer disease [PMID: 11378846 ]

SERPINA3

- increased expression of SERPINA3 in brain correlates with Alzheimer disease [PMID: 9688331 ]
- increased expression of SERPINA3 in brain correlates with amyloidosis associated with Alzheimer disease [PMID: 1702930 ]
- increased expression of SERPINA3 in cerebrospinal fluid correlates with Alzheimer disease [PMID: 17761554 ]
- increased expression of SERPINA3 in plasma correlates with increased severity of cognition disorders associated with Alzheimer disease [PMID: 12509851 ]
- increased expression of SERPINA3 in serum correlates with Alzheimer disease [PMID: 1387300 ]
- increased expression of SERPINA3 in serum correlates with dementia associated with Alzheimer disease [PMID: 8157733 ]
- polymorphism in the SERPINA3 gene correlates with decreased occurrence of disease susceptibility associated with Alzheimer disease [PMID: 11941486 ]

CCL2

- increased expression of CCL2 in astrocytes may correlate with early stage or low grade form of Alzheimer disease [PMID: 11424194 ]
- increased expression of CCL2 in astrocytes may correlate with inflammation associated with Alzheimer disease [PMID: 11424194 ]
- increased expression of CCL2 in brain correlates with inflammation associated with Alzheimer disease [PMID: 11754990 ]

GRIN2A

- decreased expression of GRIN2A in frontal cortex correlates with Alzheimer disease [PMID: 11038245 ]
- decreased expression of GRIN2A in hippocampus correlates with Alzheimer disease [PMID: 12127670 ]

RELN

- increased expression of RELN in frontal cortex correlates with Alzheimer disease [PMID: 16567613 ]
- increased expression of RELN protein correlates with Alzheimer disease [PMID: 12645087 ]

CTSB

- Our data indicate that CGA-activated microglia can trigger neuronal apoptosis and that this may be mediated through the secretion of cathepsin B.  [PMID:11238732]
- Our results suggest that cathepsin B plays novel roles in the metabolism of APP and that an inhibition of APP phosphorylation is an attractive therapeutic target for Alzheimer's disease. [PMID:21746863]
- We hypothesize that cathepsin B levels can discriminate persons with AD or MCI from healthy controls.[PMID:20930303]

SORL1

- decreased expression of SORL1 in brain correlates with increased neuron apoptosis associated with Alzheimer disease [PMID: 16452683 ]
- decreased expression of SORL1 in brain correlates with increased severity of amyloidosis associated with Alzheimer disease [PMID: 17589324 ]
- polymorphism in the SORL1 gene correlates with increased occurrence of late onset form of Alzheimer disease [PMID: 17220890 ]

TGFB1

- increased expression of TGFB1 in brain correlates with cerebral amyloid angiopathy associated with Alzheimer disease [PMID: 9335500 ]
- increased expression of TGFB1 in cerebrospinal fluid may correlate with abnormal beta-amyloid metabolic process associated with Alzheimer disease [PMID: 15331151 ]
- polymorphism in the TGFB1 gene may correlate with late onset form of Alzheimer disease [PMID: 10914688 ]

BAX

- decreased expression of BAX in hippocampus correlates with increased neurons survival associated with Alzheimer disease [PMID: 9098548 ]

IL1B

- increased expression of IL1B in blood correlates with Alzheimer disease [PMID: 14975597 ]
- increased expression of IL1B in brain correlates with Alzheimer disease [PMID: 15176485 ]
- increased expression of IL1B in cerebrospinal fluid correlates with increased incidence of non-familial form of Alzheimer disease [PMID: 8787820 ]
- increased expression of IL1B in cerebrospinal fluid may correlate with Alzheimer disease [PMID: 7693756 ]
- increased expression of IL1B in hippocampus correlates with Alzheimer disease [PMID: 7838374 ]
- single nucleotide polymorphism in the IL1B gene correlates with disease progression associated with Alzheimer disease [PMID: 15212826 ]
- single nucleotide polymorphism in the IL1B gene may correlate with genetic predisposition to disease associated with Alzheimer disease [PMID: 15653174 ]

GSK3b

- increased expression of GSK3B in brain correlates with Alzheimer disease [PMID: 10486203 ]
- increased expression of GSK3B in leukocytes correlates with Alzheimer disease [PMID: 15555766 ]
- increased expression of GSK3B in neurons may correlate with increased protein amino acid phosphorylation associated with Alzheimer disease [PMID: 8930358 ]
- increased phosphorylation of GSK3B correlates with Alzheimer disease [PMID: 15126504 ]

TUBA1B

- The alpha-tubulin mRNA was strongly expressed in neurons in the gyrus dentatus,[PMID: 7479369]

PTGS2

- increased expression of PTGS2 in hippocampus correlates with Alzheimer disease [PMID: 12770691 ]
- increased expression of PTGS2 in neurons correlates with Alzheimer disease [PMID: 12770691 ]
- increased expression of PTGS2 in pyramidal cells correlates with Alzheimer disease [PMID: 10412020 ]

MAPT

- abnormal expression of MAPT epitope correlates with disease progression associated with Alzheimer disease [PMID: 15356202 ]
- abnormal expression of MAPT epitope correlates with neurofibrillary tangles associated with Alzheimer disease [PMID: 15356202 ]
- abnormal mRNA splicing of MAPT correlates with non-familial form of Alzheimer disease [PMID: 16371011 ]
- abnormal phosphorylation of MAPT correlates with Alzheimer disease [PMID: 1826835 ]
- alternative form of MAPT mRNA correlates with disease progression associated with Alzheimer disease [PMID: 16478530 ]
- increased expression of MAPT in cerebrospinal fluid correlates with disease progression associated with Alzheimer disease [PMID: 16843497 ]
- mislocalization of MAPT protein correlates with Alzheimer disease [PMID: 1707726 ]
- nitration of MAPT may correlate with Alzheimer disease [PMID: 17050703 ]
- polymorphism in the MAPT gene correlates with early onset form of Alzheimer disease [PMID: 15106853 ]
- polymorphism in the MAPT locus correlates with disease susceptibility associated with Alzheimer disease [PMID: 16000317 ]
- single nucleotide polymorphism in the MAPT locus may correlate with Alzheimer disease [PMID: 17179995 ]

IL6

- decreased secretion of IL6 correlates with increased severity of dementia associated with Alzheimer disease [PMID: 12928049 ]
- increased expression of IL6 in blood correlates with Alzheimer disease [PMID: 12928051 ]
- increased expression of IL6 in brain correlates with inflammation associated with Alzheimer disease [PMID: 11754990 ]
- increased expression of IL6 in cerebrospinal fluid correlates with dementia associated with Alzheimer disease [PMID: 10865077 ]
- increased expression of IL6 in cerebrospinal fluid correlates with increased acute-phase response associated with Alzheimer disease [PMID: 8787820 ]
- increased expression of IL6 in cerebrospinal fluid correlates with non-familial form of Alzheimer disease [PMID: 8787820 ]
- increased expression of IL6 in microglia may correlate with Alzheimer disease [PMID: 11424194 ]
- increased expression of IL6 in plasma correlates with increased incidence of disease susceptibility associated with Alzheimer disease [PMID: 11992567 ]
- increased expression of IL6 in plasma may correlate with inflammation associated with Alzheimer disease [PMID: 10674995 ]
- increased secretion of IL6 correlates with inflammation associated with Alzheimer disease [PMID: 11754990 ]
- polymorphism in the IL6 gene correlates with decreased occurrence of non-familial form of Alzheimer disease [PMID: 10319892 ]

IL10

- increased expression of IL10 in microglia may correlate with Alzheimer disease [PMID: 12404514 ]
- polymorphism in the IL10 promoter correlates with increased incidence of non-familial form of Alzheimer disease [PMID: 14746878 ]
- single nucleotide polymorphism in the IL10 promoter correlates with Alzheimer disease [PMID: 15212825 ]

A2M

- increased expression of A2M in brain correlates with Alzheimer disease [PMID: 1712317 ]
- increased expression of A2M in plasma correlates with more severe form of Alzheimer disease [PMID: 17071923 ]
- increased expression of A2M protein may correlate with increased cell death associated with Alzheimer disease [PMID: 10072300 ]
- polymorphism in the A2M gene correlates with increased incidence of disease susceptibility associated with Alzheimer disease [PMID: 15931081 ]

NTRK1

- decreased expression of NTRK1 in caudate nucleus correlates with Alzheimer disease [PMID: 10993689 ]
- decreased expression of NTRK1 in frontal cortex correlates with Alzheimer disease [PMID: 9225742 ]
- decreased expression of NTRK1 in neurons correlates with Alzheimer disease [PMID: 9051746 ]
- decreased expression of NTRK1 in parietal cortex correlates with Alzheimer disease [PMID: 9507943 ]
- decreased expression of NTRK1 in telencephalon correlates with Alzheimer disease [PMID: 9225742 ]
- Decreased TrkA gene expression in cholinergic neurons of the striatum and basal forebrain of patients with Alzheimer's disease [PMID:9184126]

NGF

- increased expression of NGF in frontal cortex correlates with Alzheimer disease [PMID: 8501520 ]
- increased expression of NGF in occipital cortex correlates with Alzheimer disease [PMID: 8501520 ]

PSEN1

- ]
- decreased expression of PSEN1 in hippocampus correlates with Alzheimer disease [PMID: 9067452 ]
- decreased protein binding of PSEN1 correlates with Alzheimer disease [PMID: 10754226 ]
- deletion mutation in the PSEN1 promoter correlates with increased occurrence of early onset form of Alzheimer disease [PMID: 10655540 ]
- increased expression of PSEN1 in brain correlates with Alzheimer disease [PMID: 17586478 ]
- increased expression of PSEN1 mRNA correlates with Alzheimer disease [PMID: 18256261 ]
- increased expression of PSEN1 protein correlates with cerebral amyloid angiopathy associated with Alzheimer disease [PMID: 9573389 ]
- mutation in the PSEN1 gene correlates with familial form of Alzheimer disease [PMID: 15776278 ]
- mutation in the PSEN1 gene correlates with increased severity of neurofibrillary tangles associated with Alzheimer disease [PMID: 15946688 ]
- mutation in the PSEN1 gene may correlate with abnormal protein amino acid dephosphorylation associated with Alzheimer disease [PMID: 8805118 ]
- polymorphism in the PSEN1 locus correlates with increased occurrence of late onset form of Alzheimer disease [PMID: 8596269 ]
- single nucleotide polymorphism in the PSEN1 gene correlates with disease susceptibility associated with Alzheimer disease [PMID: 16938285 ]

ACHE

- abnormal expression of ACHE in cerebrospinal fluid correlates with Alzheimer disease [PMID: 9351648 ]
- abnormal glycosylation of ACHE correlates with Alzheimer disease [PMID: 10098867 ]
- decreased acetylcholinesterase activity of ACHE correlates with Alzheimer disease [PMID: 15854764 ]
- decreased acetylcholinesterase activity of ACHE correlates with sleep-wake transition disorders associated with Alzheimer disease [PMID: 17539952 ]
- decreased expression of ACHE in brain correlates with Alzheimer disease [PMID: 9269216  , 10939570 ]
- decreased expression of ACHE in brain correlates with late onset form of Alzheimer disease [PMID: 10939570 ]
- decreased expression of ACHE in cerebral cortex correlates with Alzheimer disease [PMID: 7830069 ]
- decreased expression of ACHE in lymphocytes correlates with non-familial form of Alzheimer disease [PMID: 8195795 ]
- decreased expression of ACHE in neurons correlates with Alzheimer disease [PMID: 1508295 ]
- decreased expression of ACHE in pyramidal cells correlates with nerve degeneration associated with Alzheimer disease [PMID: 1508295 ]
- decreased plasma membrane localization of ACHE correlates with Alzheimer disease [PMID: 1491735 ]
- increased expression of ACHE in cerebrospinal fluid correlates with dementia associated with Alzheimer disease [PMID: 1671469 ]
- increased expression of ACHE in frontal cortex correlates with Alzheimer disease [PMID: 9681463 ]

SNCA

- increased expression of SNCA in brain correlates with Alzheimer disease [PMID: 8546207 ]
- increased expression of SNCA in frontal cortex correlates with early stage or low grade form of Alzheimer disease [PMID: 8782917 ]
- increased expression of SNCA in neurons correlates with neurofibrillary tangles associated with Alzheimer disease [PMID: 10727692 ]
- increased expression of SNCA in neurons correlates with tauopathies associated with Alzheimer disease [PMID: 10727692 ]
- increased inclusion body localization of SNCA correlates with Lewy body disease associated with Alzheimer disease [PMID: 11117482 ]
- increased nitration of SNCA correlates with Lewy body disease associated with Alzheimer disease [PMID: 11062131 ]

LRP8

- polymorphism in the LRP8 gene may correlate with Alzheimer disease [PMID: 12399018 ]
- We speculated that another member of this LDL receptor family, LRP8 (also called apolipoprotein E receptor 2 or ApoER2), which is predominantly expressed in brain, might be associated with Alzheimer's disease. [PMID: 12399018]
- We also found that expression of LRP8 increased APP association with lipid rafts and increased gamma-secretase activity, both of which might contribute to increased Abeta production. [PMID: 17620134]

PIN1

- abnormal expression of PIN1 protein correlates with early stage or low grade form of Alzheimer disease [PMID: 14572447 ]
- abnormal tau protein binding of PIN1 correlates with Alzheimer disease [PMID: 10391244 ]
- increased oxidation of PIN1 correlates with disease progression associated with Alzheimer disease [PMID: 16466929 ]
- mislocalization of PIN1 protein correlates with Alzheimer disease [PMID: 10391244 ]

F2

- abnormal expression of F2 protein may correlate with nerve degeneration associated with Alzheimer disease [PMID: 8544905 ]
- mislocalization of F2 protein correlates with Alzheimer disease [PMID: 1491781 ]

IL2

- increased expression of IL2 in hippocampus correlates with Alzheimer disease [PMID: 7838374 ]
- increased secretion of IL2 correlates with more severe form of Alzheimer disease [PMID: 8034754 ]

CTNND2

- abnormal protein binding of CTNND2 may correlate with early onset form of Alzheimer disease [PMID: 11447843 ]
- CTNND2 based adhesion enhances Abeta release and decreases Abeta42/40 ratio [PMID: 19046403]

ACE

- increased expression of ACE in parietal cortex correlates with Alzheimer disease [PMID: 11445253 ]
- increased expression of ACE in temporal cortex correlates with Alzheimer disease [PMID: 1664329 ]
- polymorphism in the ACE gene correlates with Alzheimer disease [PMID: 10643899 ]

PRNP

- polymorphism in the PRNP gene correlates with increased occurrence of disease susceptibility associated with Alzheimer disease [PMID: 12601712 ]
- polymorphism in the PRNP gene correlates with increased occurrence of early onset form of Alzheimer disease [PMID: 15277640 ]

POMC

- abnormal secretion of POMC correlates with Alzheimer disease [PMID: 1846870 ]
- alternative form of POMC protein correlates with Alzheimer disease [PMID: 2974929 ]
- decreased expression of POMC in cerebrospinal fluid correlates with dementia associated with Alzheimer disease [PMID: 2840605 ]

BACE1

- increased expression of BACE1 protein correlates with Alzheimer disease [PMID: 12112088 ]
- polymorphism in the BACE1 gene correlates with increased occurrence of disease susceptibility associated with Alzheimer disease [PMID: 15931081 ]

DLG4

- decreased expression of DLG4 in brain may correlate with Alzheimer disease [PMID: 15979210 ]
- decreased synapse localization of DLG4 correlates with Alzheimer disease [PMID: 15509549 ]

NGFR

- decreased expression of NGFR in hippocampus correlates with increased occurrence of early onset form of Alzheimer disease [PMID: 1436650 ]
- decreased expression of NGFR in neurons correlates with Alzheimer disease [PMID: 10683291 ]
- decreased expression of NGFR in telencephalon correlates with Alzheimer disease [PMID: 10683291 ]
- increased expression of NGFR in cerebral cortex correlates with Alzheimer disease [PMID: 1309947 ]

CLU

- alternative form of CLU protein correlates with Alzheimer disease [PMID: 14559363 ]
- decreased glycosylation of CLU correlates with increased beta-amyloid formation associated with Alzheimer disease [PMID: 16490286 ]
- increased expression of CLU in cerebrospinal fluid correlates with increased microtubule cytoskeleton organization associated with Alzheimer disease [PMID: 16490286 ]
- increased expression of CLU in frontal cortex correlates with Alzheimer disease [PMID: 9878186 ]
- increased expression of CLU in hippocampus correlates with Alzheimer disease [PMID: 1702645 ]

CDK5

- increased expression of CDK5 in neurons correlates with Alzheimer disease [PMID: 10620662 ]
- increased expression of CDK5 in neurons correlates with early stage or low grade form of Alzheimer disease [PMID: 9666145 ]
- single nucleotide polymorphism in the CDK5 gene correlates with disease susceptibility associated with Alzheimer disease [PMID: 15917097 ]

TP53

- abnormal expression of TP53 in fibroblasts correlates with defective response to hydrogen peroxide associated with Alzheimer disease [PMID: 12118068 ]
- abnormal expression of TP53 in fibroblasts correlates with non-familial form of Alzheimer disease [PMID: 12118068 ]
- increased expression of TP53 in brain correlates with increased DNA fragmentation during apoptosis associated with Alzheimer disease [PMID: 9395128 ]

GRK5

- Recent studies have indicated the possible involvement of GRK, primarily altered GRK2 and GRK5, dysfunction in the pathogenesis of AD. [20730384]
- Altogether, these findings indicate that GRK5 deficiency accelerates β-amyloidogenic APP processing and Aβ accumulation in APPsw mice via impaired cholinergic activity and that presynaptic M2 hyperactivity is the specific target for eliminating the pathologic impact of GRK5 deficiency. [21041302]
- GRK5 alteration may further increase beta amyloid production in Alzheimer's disease and exaggerates brain inflammation, possibly even the basal forebrain cholinergenic degeneration [20730384]

PSEN2

- alternative form of PSEN2 mRNA correlates with abnormal pyramidal cells structure associated with Alzheimer disease [PMID: 12133587 ]
- decreased expression of PSEN2 in forebrain correlates with Alzheimer disease [PMID: 8918895 ]
- decreased expression of PSEN2 in hippocampus correlates with early stage or low grade form of Alzheimer disease [PMID: 10891593 ]
- increased expression of PSEN2 in glial cells correlates with Alzheimer disease [PMID: 9067452 ]
- missense mutation in the PSEN2 gene correlates with idiopathic form of Alzheimer disease [PMID: 9384602 ]

APOE

- abnormal beta-amyloid metabolic process associated with APOE correlates with increased occurrence of genetic predisposition to disease associated with Alzheimer disease [PMID: 10939571 ]
- decreased expression of APOE in cerebrospinal fluid correlates with Alzheimer disease [PMID: 10823584 ]
- increased expression of APOE in plasma correlates with Alzheimer disease [PMID: 10208564 ]
- increased expression of APOE mRNA correlates with Alzheimer disease [PMID: 17586478 ]
- increased expression of APOE protein may correlate with decreased neurons function associated with Alzheimer disease [PMID: 10694577 ]
- polymorphism in the APOE gene correlates with decreased response to oxidative stress associated with Alzheimer disease [PMID: 10671320 ]
- polymorphism in the APOE gene correlates with early onset form of Alzheimer disease [PMID: 17485647 ]
- polymorphism in the APOE gene correlates with increased occurrence of delusions associated with Alzheimer disease [PMID: 16841077 ]
- polymorphism in the APOE gene correlates with increased occurrence of depression associated with Alzheimer disease [PMID: 17337010 ]
- polymorphism in the APOE gene correlates with increased occurrence of early onset form of Alzheimer disease [PMID: 8786847  , 11311499 ]
- polymorphism in the APOE gene correlates with increased occurrence of genetic predisposition to disease associated with Alzheimer disease [PMID: 17280645  , 17498878  , 17553421 ]
- polymorphism in the APOE gene correlates with increased occurrence of irritable mood associated with Alzheimer disease [PMID: 16841077 ]
- polymorphism in the APOE promoter correlates with increased occurrence of genetic predisposition to disease associated with Alzheimer disease [PMID: 12105308 ]
- single nucleotide polymorphism in the APOE enhancer correlates with increased occurrence of cognition disorders associated with Alzheimer disease [PMID: 17613540 ]
- single nucleotide polymorphism in the APOE gene correlates with late onset form of Alzheimer disease [PMID: 17317784 ]

TTR

- abnormal expression of TTR in cerebrospinal fluid correlates with Alzheimer disease [PMID: 11973456 ]
- decreased expression of TTR in cerebrospinal fluid correlates with Alzheimer disease [PMID: 9559653 ]

LDLR

- Our findings suggest that LDLR gene may be associated with AD risk and is a CSF biomarkers, especially in women.[PMID:17239995]
- increasing glial LDLR levels may promote Aβ degradation within the brain.[22383525]
- Therefore, these results identify LDLR as a receptor that mediates Aβ uptake and clearance by astrocytes, and provide evidence that increasing glial LDLR levels may promote Aβ degradation within the brain. [PMID: 22383525]
- These findings suggest that increasing LDLR levels may represent a novel AD treatment strategy.[PMID: 20005821]
- We found that over-expression of APP may cause increase in both LDLR mRNA and protein levels in APP transfected H4 neuroglioma cells compared to H4 controls.[ 20049331]

GRM1

- decreased expression of GRM1 in brain may correlate with dementia associated with Alzheimer disease [PMID: 15949941 ]
- These data suggest that group II mGluR may trigger synaptic activation of all three secretases and that suppression of group II mGluR signaling may be a therapeutic strategy for selectively reducing synaptic generation of Abeta(42). [PMID:20237257]
- induced stimulation of GRM1 protein may correlate with abnormal signal transduction associated with Alzheimer disease [PMID: 12054503 ]

APP

- abnormal expression of APP in cerebrospinal fluid correlates with non-familial form of Alzheimer disease [PMID: 17049739 ]
- abnormal processing of APP correlates with cognition disorders associated with Alzheimer disease [PMID: 17356877 ]
- abnormal processing of APP correlates with familial form of Alzheimer disease [PMID: 16752394 ]
- abnormal processing of APP correlates with non-familial form of Alzheimer disease [PMID: 17049739 ]
- alternative form of APP protein correlates with decreased neurons survival associated with Alzheimer disease [PMID: 8752124 ]
- alternative form of APP protein correlates with dementia associated with Alzheimer disease [PMID: 1642473 ]
- alternative form of APP protein may correlate with decreased neurons survival associated with Alzheimer disease [PMID: 8752124 ]
- APP map position correlates with late onset form of Alzheimer disease [PMID: 11500807 ]
- decreased expression of APP in cerebrospinal fluid correlates with Alzheimer disease [PMID: 11552007  , 12486489 ]
- decreased expression of APP in plasma correlates with non-familial form of Alzheimer disease [PMID: 8347821 ]
- decreased expression of APP mRNA correlates with Alzheimer disease [PMID: 8294927 ]
- decreased extracellular region localization of APP correlates with abnormal protein processing associated with Alzheimer disease [PMID: 7957195 ]
- decreased secretion of APP correlates with abnormal amyloid precursor protein metabolic process associated with Alzheimer disease [PMID: 9872930 ]
- deletion mutation in the APP gene correlates with non-familial form of Alzheimer disease [PMID: 11238715 ]
- hypomethylation of the APP gene correlates with Alzheimer disease [PMID: 8746452 ]
- increased endosome localization of APP correlates with non-familial form of Alzheimer disease [PMID: 15465622 ]
- increased expression of APP in brain correlates with Alzheimer disease [PMID: 3159021 ]
- increased expression of APP in cerebrospinal fluid correlates with late onset form of Alzheimer disease [PMID: 17366635 ]
- increased expression of APP in frontal cortex correlates with nerve degeneration associated with Alzheimer disease [PMID: 1331685 ]
- increased expression of APP in lens correlates with increased occurrence of cataract associated with Alzheimer disease [PMID: 12699953 ]
- increased expression of APP in monocytes correlates with abnormal protein processing associated with Alzheimer disease [PMID: 10588572 ]
- increased expression of APP in plasma correlates with increased occurrence of brain infarction associated with Alzheimer disease [PMID: 16401840 ]
- increased expression of APP in temporal cortex correlates with Alzheimer disease [PMID: 17586478 ]
- increased expression of APP mutant protein correlates with autosomal dominant form of Alzheimer disease [PMID: 16432153 ]
- increased expression of APP mutant protein correlates with early onset form of Alzheimer disease [PMID: 12787077 ]
- increased expression of APP mutant protein correlates with familial form of Alzheimer disease [PMID: 16432153 ]
- increased extracellular region localization of APP may correlate with non-familial form of Alzheimer disease [PMID: 11238715 ]
- increased oxidation of APP correlates with Alzheimer disease [PMID: 16816122 ]
- increased presence of APP autoimmune antibody correlates with Alzheimer disease [PMID: 15212827 ]
- increased presence of APP autoimmune antibody correlates with dementia associated with Alzheimer disease [PMID: 17477976 ]
- increased protein binding of APP correlates with familial form of Alzheimer disease [PMID: 12054732 ]
- increased proteolysis of APP correlates with abnormal protein processing associated with Alzheimer disease [PMID: 7745619 ]
- increased proteolysis of APP correlates with Alzheimer disease [PMID: 8025536 ]
- increased proteolysis of APP correlates with familial form of Alzheimer disease [PMID: 7523115 ]
- missense mutation in the APP gene correlates with abnormal cytoskeleton organization associated with Alzheimer disease [PMID: 1465214 ]
- missense mutation in the APP gene correlates with abnormal protein processing associated with Alzheimer disease [PMID: 11528419 ]
- missense mutation in the APP gene correlates with autosomal dominant form of Alzheimer disease [PMID: 12552037 ]
- missense mutation in the APP gene correlates with decreased Purkinje cells survival associated with Alzheimer disease [PMID: 9171327 ]
- missense mutation in the APP gene correlates with early onset form of Alzheimer disease [PMID: 9328472  , 12552037 ]
- missense mutation in the APP gene correlates with increased incidence of early onset form of Alzheimer disease [PMID: 8247223 ]
- missense mutation in the APP gene correlates with increased protein secretion associated with Alzheimer disease [PMID: 7957938 ]
- missense mutation in the APP gene correlates with stroke associated with Alzheimer disease [PMID: 12163376 ]
- mutation in the APP gene correlates with increased severity of neurofibrillary tangles associated with Alzheimer disease [PMID: 15946688 ]
- mutation in the APP gene may correlate with abnormal protein processing associated with Alzheimer disease [PMID: 12395079 ]
- mutation in the APP promoter correlates with Alzheimer disease [PMID: 16685645 ]
- polymorphism in the APP gene correlates with early onset form of Alzheimer disease [PMID: 16423463 ]
- polymorphism in the APP promoter correlates with late onset form of Alzheimer disease [PMID: 16243604 ]
- single nucleotide polymorphism in the APP promoter correlates with genetic predisposition to disease associated with Alzheimer disease [PMID: 17325276 ]

APOA1

- abnormal expression of APOA1 in cerebrospinal fluid correlates with Alzheimer disease [PMID: 14559363 ]
- abnormal folding of APOA1 may correlate with abnormal protein processing associated with Alzheimer disease [PMID: 7639323 ]
- decreased expression of APOA1 in serum correlates with increased severity of dementia associated with Alzheimer disease [PMID: 10794845 ]
- increased expression of APOA1 in cerebrospinal fluid correlates with Alzheimer disease [PMID: 10828089 ]

PPARG

- abnormal expression of PPARG protein may correlate with Alzheimer disease [PMID: 9920782 ]
- PPARG is expressed in brains of Alzheimer's Disease (AD) patients [PMID: 10685726]
- PPAR-gamma expression was selectively increased [PMID: 16873964]
