# Supplementary material for: Linking hypothetical knowledge patterns to disease molecular signatures for biomarker discovery in Alzheimer’s disease
Source: Genome Med. 2014 Dec 3;6(11):97. doi: 10.1186/s13073-014-0097-z (PMC4256903; doi:10.1186/s13073-014-0097-z)
Supplement: Additional file 5: — Description of annotation guidelines followed to construct a PPi network of AD by gathering evidences existing in literature. [file 13073_2014_97_MOESM5_ESM.docx]

**Annotation guidelines for curation of protein-protein interaction data**

The goal of this exercise is to extract sentences (serving as evidences) existing in literature, which represents relevant protein-protein interaction (PPi) information.

Results of experiments performed to verify a particular PPi are often conveyed in scientific publications. We aim to utilize this wealth of available pubic data and extract PPi based information specific to a particular disease domain leading to the generation of a disease specific protein interaction network.

A Machine learning approach is used for initial extraction of sentences existing in literature related to particular disease. Since we want to extract all possible information existing in literature so the automated machine learning approach was optimized to generate maximum recall. Figure 1 shows the output sentences generated by following a machine learning approach.


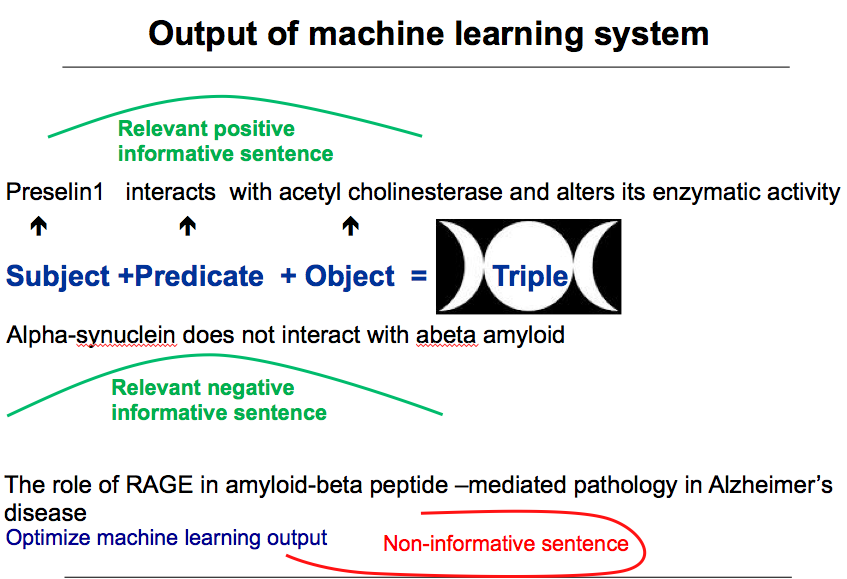


*Figure1: Sentences extracted from biological literature using a Machine learning approach trained for relationship extraction.*

Now as shown in Figure1, a automated work flow can also generate false positives. So, for the construction of a accurate disease specific interaction network "human curation" is necessary.

**Task**

You have been provided with sentences (output of a machine learning approach) which are believed to represent Protein-protein interaction(PPi) .

**Protein–protein interactions** occur when two or more [proteins](http://en.wikipedia.org/wiki/Protein) bind together, often to carry out their [biological function](http://en.wikipedia.org/wiki/Function_(biology)).

Some examples:

"Within this complex, LGI1 binds selectively to a neuronal specific membrane protein, ADAM22 (a disintegrin and metalloprotease)".

"Presenilin-1 interacts with plakoglobin and enhances plakoglobin-Tcf-4 association."

Your task is to manually cross check the output of the automated approach (sentences) and see weather they really represent a protein- protein interaction or not (Specific to the disease assigned to you) and if they do represent (PPi) then further annotate them with the below mentioned concepts.


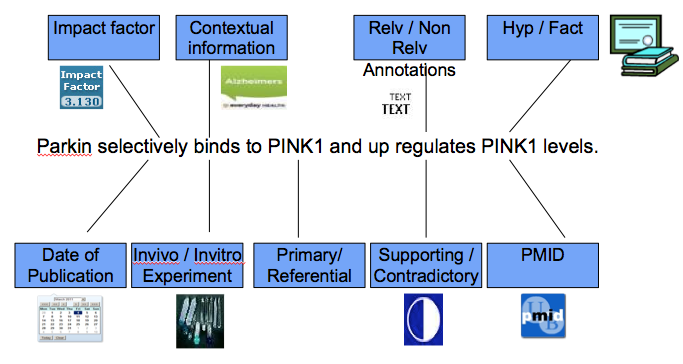


*Figure 2: Various features for which each sentence representing
valid PPI has been annotated*

You can use a excel sheet with columns to do all your annotations with features like:

contexual information, date of publication, Journal name, PMID, Impact factor of journal, Fact/Hypothesis, supporting/contradictory evidence, Invivo/invitro evidence.

Let go through each of these features in detail:

**Task 1:** Read the sentence carefully and determine weather this represent a PPi or not (relevance). If this represent PPi then assign a label 1 in front of it or if it does not represent PPi assign label 0 to it(If you assign a lable 0 to a sentence which you think does not contain PPi then you need not to do any further annotations for this sentence)

**Note:** Some time the sentence does not represent PPI but contains usefull information which we call as knowledge . Example:

**P-glycoprotein, multi-drug resistance associated protein and major vault protein contribute to the pathogenesis of refractory epilepsy.**

The sentence does not represent PPi but surely represent useful information about proteins that contribute to pathogenesis of refractory epilepsy.

All such sentences should be assigned a label 3 infront of them which means they represent knowledge.

**Just for your information:** Some times it may be difficult to classify what to consider knowledge /what not . In this case ask your self a question is this sentence helping me know some insights into the disease mechanism or can this information be useful for drug discovery purposes. Anything which is useful for us in getting new insight into the disease is knowledge for us.

**Task 2:** Determine weather information represented in the PPI really belongs to the disease assigned to you(Contextual information).

Suppose you are annotating a corpus of Alzheimer PPI’s .Sometimes what happens in abstracts is that there is just mention of Alzheimer as a word in the abstract and the whole abstract is taking about normal brain phenomenon.

So you have to make sure that the sentence is definitely taken from abstract which is taking about alzheimer’s disease.

**Task 3:** add PMID, Date of publication, Journal name and impact factor of the journal information to your annotation.

**Task 4:** Classify weather the sentence is a fact or a hypothesis

Example: You are living in Bonn

Nature is beautiful

Angela merkel is a German

These are all facts

Hypothesis/Speculation:

Tomorrow I may go to Koln

I don’t know if nature will also be beautiful in Sahara desert

Angela merkel might again become German chancellor

All these sentences are speculations because we are not sure if the are happening or not. Remember words like may, might, could be, suggest etc are used to represent speculation in text

So for each PPi sentence you have to check if it is a fact or speculation.

"GRK2 may hyperphosphorylates tau in tauopathies" (Hypothesis)

"Presenilin-1 interacts with plakoglobin and enhances plakoglobin-Tcf-4 association" (Fact)

**Task 5:** Check weather the interaction sentence mentioned is a supporting evidence or a contraction

If you are considering a interaction of A-B(Two proteins)

Then A interacts with B is supporting evidence

"In mouse, we found a high avidity binding of Abeta peptides to ACHE."

A does not interact with B is a contraction (opposite to supporting evidence)

"Our protein interaction experiment argues against interaction between APP and ACHE"

**Task 6:** Classify if a PPi is supported by a invivo evidence or In vitro evidence.

In this case you will have to read the full text to check weather the experiment conducted is invivo or invitro.

In Vivo experiment refers to a medical test, experiment or procedure that is done on a living organism, such as a laboratory animal or human.

In Vitro refers to experiments done within a [glass](http://www.biology-online.org/dictionary/Glass) or culture medium, observable in a [test tube](http://www.biology-online.org/dictionary/Test_tube), in an artificial environment [outside](http://www.biology-online.org/dictionary/Outside) the living organism;

Or

Biological [processes](http://www.biology-online.org/dictionary/Processes) or [reactions](http://www.biology-online.org/dictionary/Reactions) that would normally occur within an [organism](http://www.biology-online.org/dictionary/Organism) but here are made to occur in an [artificial](http://www.biology-online.org/dictionary/Artificial) [environment](http://www.biology-online.org/dictionary/Environment), i.e. A [laboratory](http://www.biology-online.org/dictionary/Laboratory)

You must also annotate the "interaction detection method" used to confirm a particular PPi existing in literature. Most cases particular PPI is backed up with multiple interaction detection methods. Please mention all of them with the interaction separated by comma.
